# Gender differences in publication rates at Canadian Society of Otolaryngology–Head and Neck Surgery annual meetings: an 11-year analysis

**DOI:** 10.1186/s40463-022-00612-7

**Published:** 2023-02-09

**Authors:** Dorsa Mavedatnia, Jennifer Payandeh, Penelope Neocleous, Jacob Davidson, Agnieszka Dzioba, Julie E. Strychowsky, M. Elise Graham

**Affiliations:** 1 Faculty of Medicine, University of Ottawa, Ottawa, ON Canada; 2 Faculty of Medicine, Queen’s University, Kingston, ON Canada; 3 Schulich School of Medicine and Dentistry, London, ON Canada; 4 Division of Pediatric Surgery, Children’s Hospital at London Health Sciences Center, Schulich School of Medicine, London, ON Canada; 5 Department of Otolaryngology-Head and Neck Surgery, Children’s Hospital at London Health Sciences Center, Schulich School of Medicine, 800 Commissioners Road E, London, ON N6A 5W9 Canada

**Keywords:** Otolaryngology, Gender, Research, Publications

## Abstract

**Background:**

Evaluating gender differences in publication rates after conference presentations is an avenue to assess women’s contributions to academic medicine. The objective of this study was to assess gender differences in publication rates, time to publication, and subspeciality of publication of abstracts presented at Canadian otolaryngology conferences over an 11-year period.

**Methods:**

Cross-sectional data was obtained from online conference schedules of annual Canadian Society of Otolaryngology–Head and Neck Surgery national meetings between 2009 and 2020. A total of 2111 abstract titles were searched in MedLine via PubMed. Gender of the first and senior author, publication status of presented work, and subspeciality of publication were extracted.

**Results:**

Of 2111 scientific abstracts presented between 2009 and 2020, female first and senior authors accounted for 29.0% and 12.8% of published abstracts, respectively. There was a significant difference in the publication rate of senior authors by gender (*p* < 0.01). Male senior authors had a 9.70% higher rate of publication compared to female senior authors. Posters with a female first author were 33.0% (OR: 0.67; 95% CI 0.49–0.91) less likely to be published compared to posters with a male first author. Similarly, posters with a female senior author were 34.0% (OR: 0.66; 95% CI 0.45–0.96) less likely to be published. There was a significant difference in discipline of publication by gender of the senior author (*p* < 0.001). Male senior authors were more likely to supervise projects in otology while female senior authors were more likely to supervise projects in education and pediatrics. The time to publication and impact factor of the journal of publication did not differ by gender.

**Conclusion:**

Gender disparities exist in the publication rates of first and senior authors at Canadian otolaryngology meetings. Female senior authors have significantly lower publication rates compared to their male colleagues and differences exist in publication rates after poster presentations. Investigation of gender gaps in academic medicine, research productivity, and publications is essential for development of a diverse, equitable, and inclusive workforce in otolaryngology.

**Graphical Abstract:**

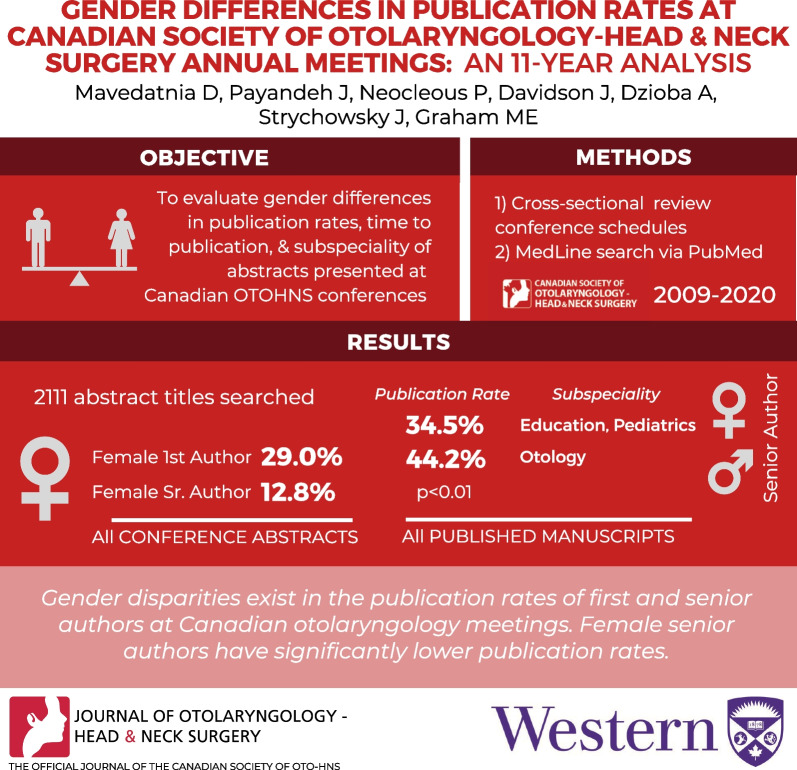

## Background

Surgical specialties, with the exception of obstetrics and gynecology, are male-dominated, with women being significantly underrepresented [1]. Women comprise approximately 50% of graduating medical students, but women account for only 10% of full professors in academic surgery [2]. When considering otolaryngology, female representation in Canadian Otolaryngology–Head and Neck Surgery (OHNS) residency programs has increased from 28.6% in 2000 to 41.9% in 2019 [3]. In 2019, females accounted for 52.9% of all OHNS residents and fellows in Canada [3], demonstrating promising improvements in gender representation within OHNS. Despite this, in the United States and Canada, females are significantly underrepresented in academic otolaryngology leadership positions, including academic chairs, and professors [3–6]. Further evidence for the widespread gender inequity in medicine is seen through research productivity and underrepresentation of women as conference speakers and research grant recipients [6–8]. At annual American Academy of Otolaryngology–Head and Neck Surgery (AAO–HNS) meetings, women represented less than 20% of submissions and more frequently presented posters as opposed to oral presentations [9].

Authorship, publication rates, and the quality and quantity of publications have important implications for a physician’s professional development and medical achievement as they impact opportunities for career advancement [10]. Presentation at conferences allows for networking and dissemination of novel research. Subsequent publications are associated with overall career success, academic career advancement [11], and play a major role in obtaining grants [12, 13]. Historically, men compromise the majority of academic otolaryngologists and have higher research productivity than their female counterparts with a greater number of publications and citations [3, 14]. The annual Canadian Society of Otolaryngology–Head and Neck Surgery (CSOHNS) meeting is the largest national Canadian otolaryngology meeting, attracting thousands of attendees every year. A previous study examined publication rates at CSOHNS meetings between 2006 and 2010 but gender was not a variable in the study [15], and limited literature exists which examines gender differences in publications in otolaryngology. While gender differences in publication rates were assessed at AAO–HNS meetings between 2000 and 2004 [9], no study has examined gender differences in presentations and publication rates at national CSOHNS meetings. Many years have passed since the prior study and given the lack of Canadian data and that representation of women in otolaryngology is expected to further increase, a current analysis of gender differences after conference presentation is warranted.

The aim of this study was to assess gender differences in authorship, publication rate, and time to publication through assessing peer-reviewed publication rates of abstracts presented at CSOHNS meetings over an 11-year period. The study also aimed to identify trends of publication rates over time and assess whether gender differences existed in publication rates based on the form of presentation (podium, poster, Poliquin resident competition).

## Methods

Ethics approval was not required as this was publicly available data. The Strengthening the Reporting of Observational Studies in Epidemiology (STROBE) reporting guideline was utilized in this study.

Cross sectional data were obtained from online conference schedules of national CSOHNS meetings that took place between 2009 and 2020 [16]. All abstract titles published in the scientific programs were retrospectively searched in MedLine via PubMed by three reviewers (DM, JP, PN) for publication. To standardize the search process, searches were initially conducted with the first author and a keyword from the abstract title. If not successful, this was followed by a search for the senior author. If still not successful, an alternate keyword was searched with the first and senior author. This process was followed before declaring an abstract unpublished. Abstracts were considered published if the study design, objective, and results were nearly identical to the published paper. If the published paper contained all the data from the abstract in addition to other data, it was considered as published material from the presented abstract. When differences were found in the title or authors of a publication, content of the abstract was compared to the published paper to determine eligibility. Once a paper was deemed as published according to these criteria, the search ceased, without expanding for multiple papers generated from the same abstract. This search strategy has been described and utilized in other studies [9, 10, 15, 17–20]. Quality control was performed with a fourth reviewer (EG). A random number generator was used to identify 10 abstracts from each year (110 total abstracts) and the results of data extraction were compared. Of the 110 abstracts that were cross-validated, 3 were found to be published by the fourth reviewer but were rated as unpublished by initial reviewers, representing an acceptable miss-rate of 2.7%.

Extracted variables included the type of presentation, subspeciality, gender of the first and senior author, presentation date, publication date, journal of publication, and if there was an authorship change for the first or senior author, resulting in an author of a different gender for the published paper. If a presentation listed only one author, it was categorized as the first author. The presentation types included: podium, poster, and Poliquin resident competition. Podium presentations are oral presentations which are given a formal time slot and 5-min in length. Poster presentations are informal and presented during poster sessions. The Poliquin resident competition is a 5-min oral presentation with submissions restricted to Canadian OHNS resident first authors and abstracts must be submitted with a completed manuscript. Subspecialities were categorized based on program abstracts and included: general otolaryngology, head and neck, otology, rhinology, pediatrics, facial and reconstructive plastic surgery, and education. The time to publication was calculated according to the presentation and publication date, with the time to publication rounded to the nearest month. Articles which were published in peer-reviewed journals prior to the annual CSOHNS meeting were provided with negative numbers.

Gender identity was coded as “male”, or “female”, or “unknown” if the author was not found. This variable was coded based on author name, and/or Google search performed using author name with/without subspecialty to find publicly available photos for reference.

### Statistical analysis

The cohort of CSOHNS presenters were characterized using descriptive statistics. Differences in gender across presentation type, discipline of presentation, and publication rate were examined using chi-square test and logistic regression with odds ratios (OR) and 95% confidence intervals (95% CI). A two-tailed alpha of 0.05 was used to determine significant differences between genders. The analysis and all statistical tests were completed using SAS Software (version 9.4; SAS Inc., Cary, NC).

## Results

### Abstract presentations at CSOHNS

Between 2009 and 2020, 2,139 total scientific abstracts were presented, consisting of 922 posters (43.1%), 191 Poliquin resident competition presentations (8.9%), and 1,026 podium presentations (48.0%). Of the abstracts presented, the gender of the first author could not be determined for 12 poster presentations and 16 podium presentations, resulting in a final sample size of 2111 abstracts.

Majority of abstracts presented at CSOHNS meetings were from male first authors (68.7%), with female first authors accounting for 31.3% of presentations. Although there was an overall greater number of male-led abstracts, there was no significant difference (*p* = 0.28) in the type of presentation across the gender of the first author, with podiums being the most common for both sexes (males: 46.9% vs. females: 49.9%), following by posters, and Poliquin presentations (Table 1). However, for senior authors, there was a significant difference in the type of presentation they were offered (*p* = 0.01), with male senior authors supervising approximately 5% more Poliquin resident competition presentation and 5% less poster presentations compared to female senior authors (Table 2).

**Table 1:** Abstract presentations and publications by the gender of the first author

	Overall	Males	Females	*p* value
	% (n)			
Presentations	N = 2111	N = 1450	N = 661	0.28
Poster	43.1 (910)	43.5 (631)	42.2 (279)	
Poliquin resident competition	9.0 (191)	9.6 (139)	7.9 (52)	
Podium	47.9 (1010)	46.9 (680)	49.9 (330)	
Publication	N = 971	N = 689	N = 282	0.46
Poster	33.8 (328)	35.0 (241)	30.9 (87)	
Poliquin resident competition	15.5 (151)	15.4 (106)	16.0 (45)	
Podium	50.7 (492)	49.6 (342)	53.1 (150)

**Table 2:** Abstract presentation and publications by the gender of the senior author

	Overall	Males	Females	*p* value
	% (n)			
Presentation	N = 2057	N = 1730	N = 327	< 0.01
Poster	43.4 (893)	42.6 (737)	47.7 (156)	
Poliquin resident competition	9.3 (191)	10.1 (175)	4.9 (16)	
Podium	47.3 (973)	47.3 (818)	47.4 (155)	
Publication	N = 878	N = 765	N = 113	0.14
Poster	34.6 (304)	34.8 (266)	33.7 (38)	
Poliquin resident competition	15.4 (135)	16.2 (124)	9.7 (11)	
Podium	50.0 (439)	49.0 (375)	56.6 (64)	

### Abstract publication at CSOHNS

**Fig. 1:**
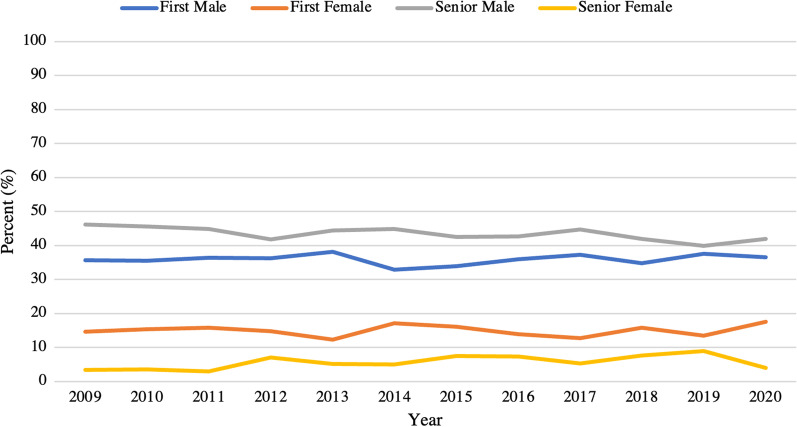
Proportion of publications by gender and first or senior author over time. No significant changes were noted over the studied time period

A total of 971 (46.0%) abstracts were published between 2009 and 2020. There was no significant difference in the proportion of abstracts published by author gender over time, with male senior authors consistently having the highest average proportion of publications (43.5%), followed by male first authors (35.9%), female first authors (15.0%), and female senior authors (5.7%) (Fig. 1).

**Fig. 2:**
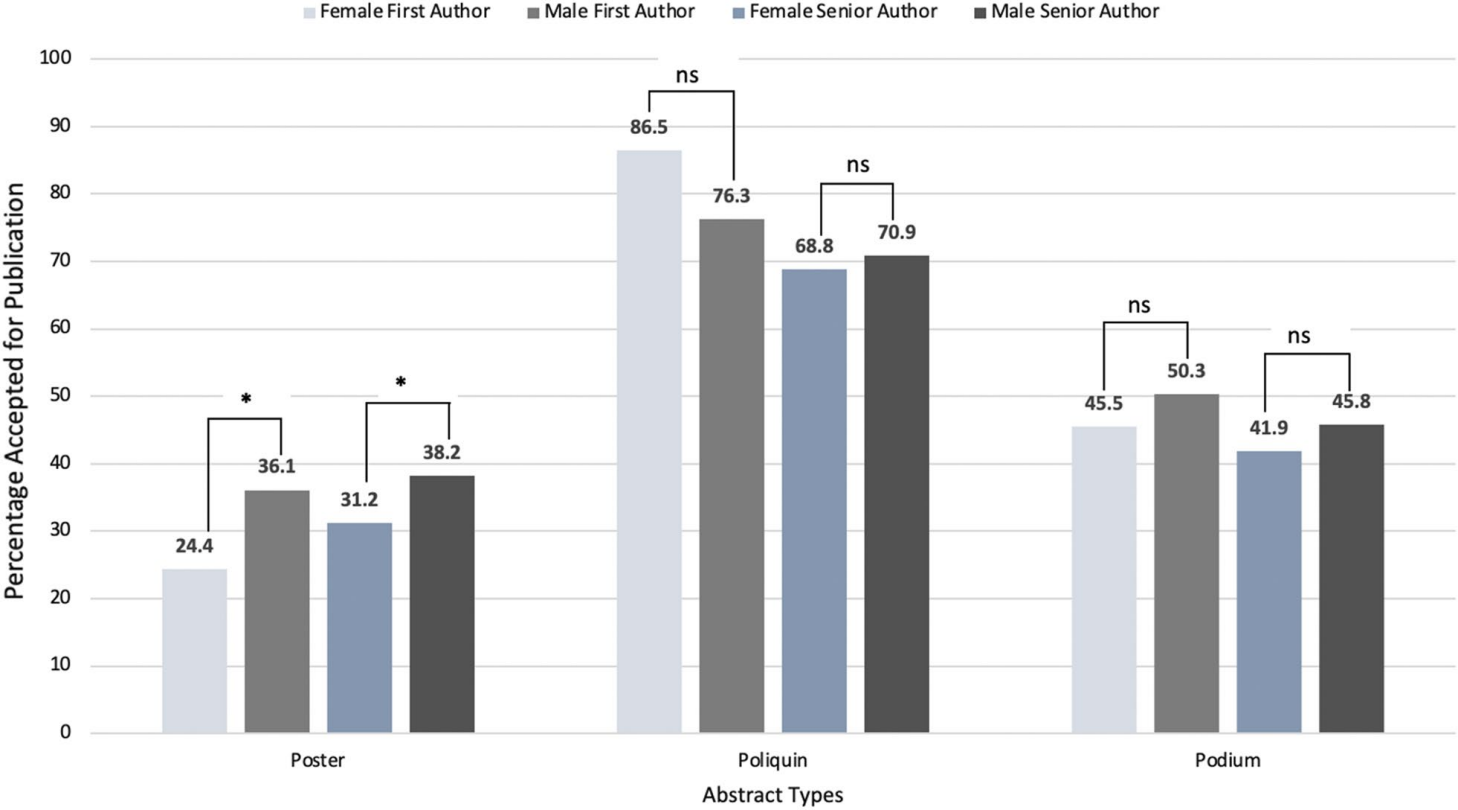
Publication rate (% published) by presentation type and author gender. Note there is a significant difference in proportion of posters leading to publication by first and senior author gender

The rate of publication for each type of presentation differed, with 79.1% of Poliquin resident competition presentations published, 48.6% of podium presentations, and 36.0% of poster presentations. The overall rate of publication for first authors was 45.9% and 48.0% for senior authors. There was no significant difference in proportion of abstracts published by subspecialty (*p* = 0.28) (Table 3) There was also no significant difference in publication rate of first authors by gender (male: 47.5%, female: 42.7%; *p* = 0.07), but there was a significant difference for senior authors. Male senior authors had almost a 10% higher rate of publication compared to female senior authors (male: 44.2% vs. female: 34.5%; *p* < 0.01) (Fig. 2).

**Table 3:** Publication rates by Otolaryngology–Head and Neck Surgery subspecialties

Author subspecialty	Published (N = 971)	Not published (N = 1164)	*p* value
	% (n)		
General	10.3 (100)	13.2 (154)	0.2784
Rhinology	9.4 (91)	10.1 (118)	
Head and Neck	30.7 (298)	27.0 (314)	
Pediatrics	10.3 (100)	10.7 (124)	
Otology	17.4 (169)	17.4 (203)	
Laryngology	3.8 (37)	4.0 (47)	
Facial/Plastics Reconstructive Surgery	4.6 (45)	4.5 (52)	
Endocrinology	5.7 (55)	4.2 (49)

Overall, only 29.0% of the total published abstracts were from female first authors. Across publication type, only 29.8% of published Poliquin presentations had a female first author, 25.4% of poster presentations, and 31.8% of published podium presentations. There was no significant difference in the likelihood of being published by gender of the first author for either Poliquin presentations (OR: 2.00; 95% CI 0.82–4.86) or podium presentations (OR: 0.92; 95% CI 0.71–1.20). However, posters with a female first author were 33.0% (OR: 0.67; 95% CI 0.49–0.91) less likely to be published compared to posters with a male first author.

**Fig. 3:**
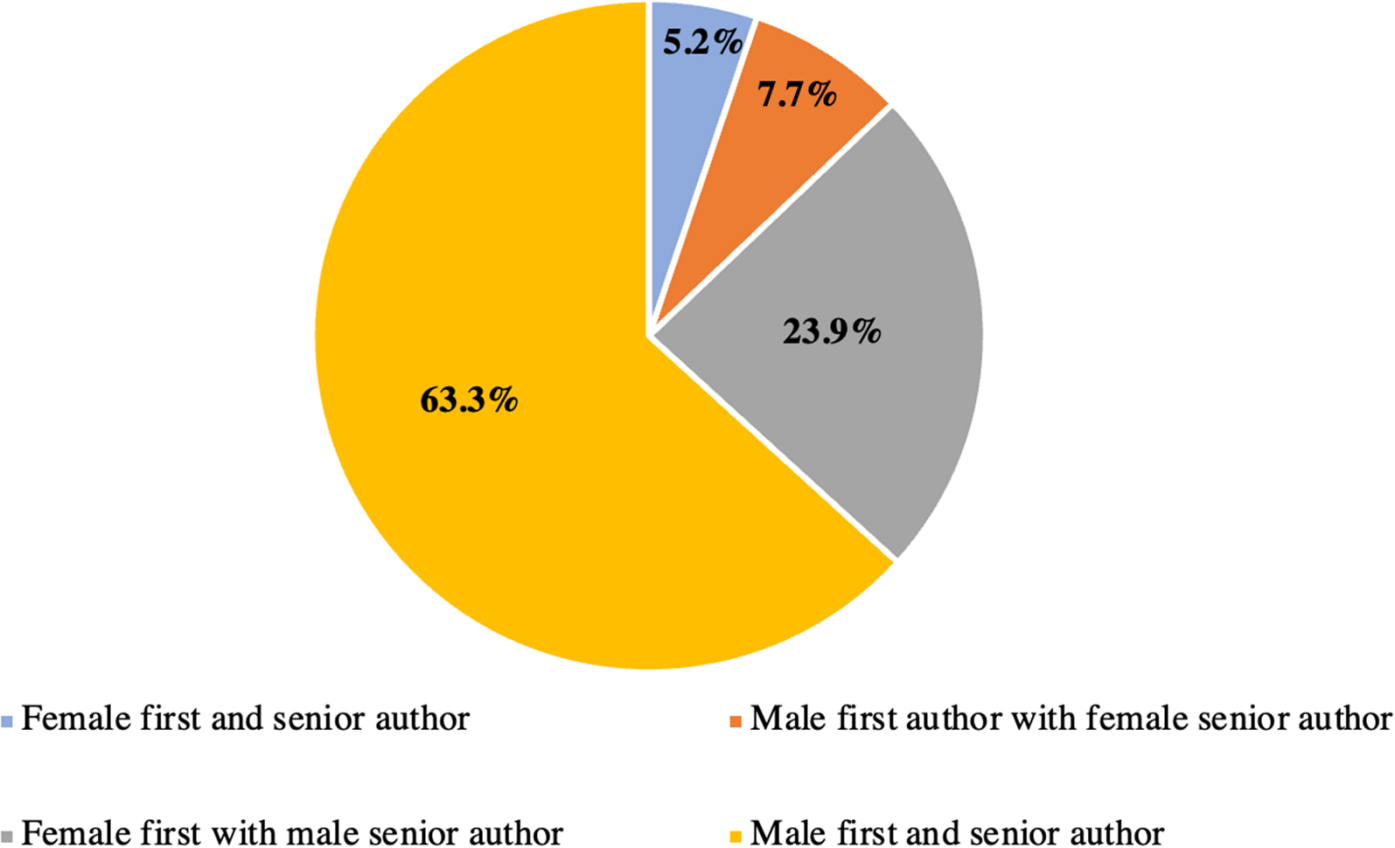
Gender representation of authors in published papers, stratified by first and senior author. Most papers arising from abstracts were authored by both a male first and senior author. Those authored by a female first and senior author made up the smallest proportion, at 5.2%

A similar trend was observed for senior authors, with female senior authors accounting for 12.8% of published abstracts. There was no difference in the likelihood of publication for Poliquin resident competition presentations (OR: 0.78; 95% CI 0.24–2.56) or podium presentations (OR: 0.80; 95% CI 0.57–1.23) by gender of the senior author. Posters with a female senior author were 34.0% (OR: 0.66; 95% CI 0.45–0.96) less likely to be published compared to posters with a male senior author. Of all abstracts published, only 5.2% were authored by both a female first and senior author, compared with 63.3% authored by a male first and senior author (Fig. 3).

**Fig. 4:**
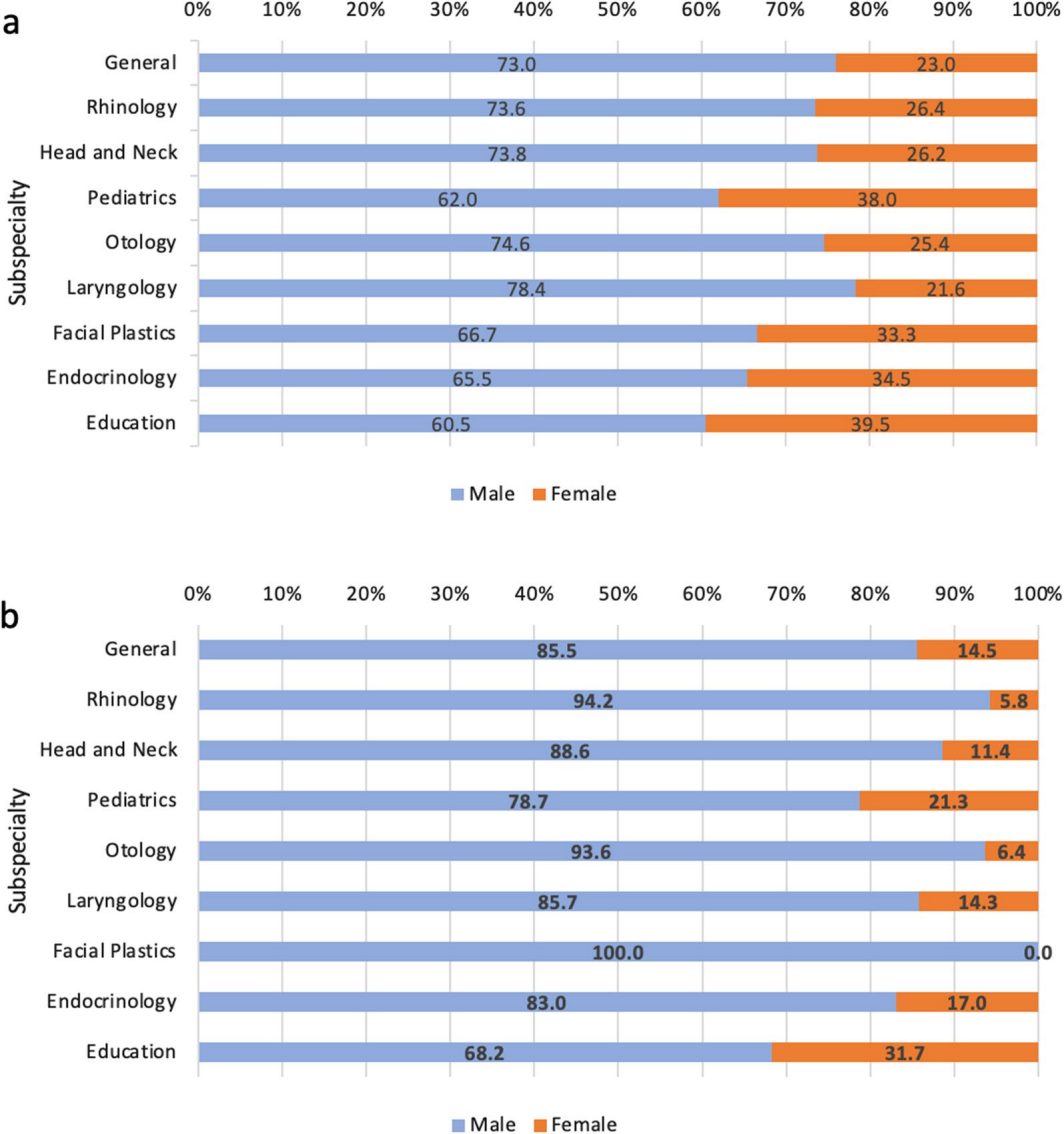
**a**, **b** Proportion of publications by subspeciality of first (**a**) and senior (**b**) author. There was no difference in subspecialty of presentation by gender of first author (*p* = 0.11), however the gender differences by subspecialty were significantly different for senior authors (*p* < 0.01)

There was no significant difference in the discipline of a published presentation across gender for the first author (*p* = 0.11) (Table 4). In contrast, there was a significant difference in the discipline of published presentation across gender of the senior author (*p* < 0.001). The three subspecialties with the greatest difference between male and female senior authors were otology, education, and pediatrics. Male senior authors supervised 10.3% more presentations in otology compared to female senior authors. Female senior authors supervised 12.9% more presentations in education and 7.8% more presentations in pediatrics compared to males (Table 5). Distribution of first and senior author gender by subspecialty is depicted in Fig. 4a, b.

**Table 4:** Subspecialties of publication by gender of first author

First author subspecialty	Males (N = 689)	Females (N = 282)	*p* value
General	10.6 (73)	9.6 (27)	0.11
Rhinology	9.7 (67)	8.5 (24)	
Head and Neck	31.9 (220)	27.7 (78)	
Pediatrics	9.0 (62)	13.5 (38)	
Otology	18.3 (126)	15.3 (43)	
Laryngology	4.2 (29)	2.8 (8)	
Facial/Plastics Reconstructive Surgery	4.4 (30)	5.3 (15)	
Endocrinology	5.2 (36)	6.7 (19)	
Education	6.7 (46)	10.6 (30)

**Table 5:** Subspecialties of publication by gender of senior author

Senior author subspecialty	Males (N = 689)	Females (N = 282)	*p* value
General	10.6 (81)	12.5 (14)	< 0.01
Rhinology	10.6 (81)	4.5 (5)	
Head and Neck	30.6 (233)	26.8 (30)	
Pediatrics	9.2 (70)	17.0 (19)	
Otology	19.2 (146)	8.9 (10)	
Laryngology	3.2 (24)	3.6 (4)	
Facial/Plastics Reconstructive Surgery	5.0 (38)	0.0 (0)	
Endocrinology	5.8 (44)	8.0 (9)	
Education	5.9 (45)	18.8 (21)

### Abstract publication rates

The median time to publication for first author was 27.9 months (IQR: 11.9–51.8) and for senior author it was 27.2 months (IQR: 10.2–50.1) (Table 6). The overall time to publication did not differ by gender of first author (*p* = 0.54) or senior author (*p* = 0.78). No significant difference was found in the median impact factor of journals for first (male: 2.9 [IQR: 2.1–2.9], females: 2.9 [IQR: 2.0–3.5]; *p* = 0.53) and senior author (male: 2.9 [IQR: 2.3–2.9], females: 2.5 [IQR: 1.7–2.9]; *p* = 0.05) by gender.

**Table 6:** Time to publication in months following presentation by first and senior author gender and presentation type

Variable	Overall	Males	Females	*p* value
	Median time to publication (interquartile range)			
First author		N = 689	N = 282	
Overall	**27.9 (11.9–51.8)**	**27.1 (11.9–51.8)**	**29.5 (11.9–52.3)**	**0.54**
Poster	30.1 (10.1.5–52.9)	27.0 (10.2–52.7)	30.1 (9.4–54.8)	0.70
Poliquin Resident Competition	22.2 (15.2–30.5)	21.8 (15.2–29.6)	24.5 (14.8–42.4)	0.24
Podium	32.2 (10.0–57.9)	32.6 (10.0–60.0)	31.6 (11.0–53.7)	0.99
Senior author		N = 765	N = 113	
Overall	**27.2 (10.2–50.1)**	**27.2 (10.2–50.1)**	**28.8 (10.1–50.1)**	**0.78**
Poster	27.3 (8.4–50.5)	27.0 (8.0–52.7)	29.5 (9.3–39.8)	0.88
Poliquin Resident Competition	22.8 (14.8–30.1)	22.7 (14.8–30.1)	23.8 (12.4–52.3)	0.54
Podium	30.5 (9.4–57.4)	32.1 (9.9–57.3)	28.8 (9.3–64.3)	0.99

The overall publication rate for first and senior authors is highlighted by the bold text

## Discussion

This is the first study to assess gender differences in authorship and publication rates at annual CSOHNS meetings. It adds to the limited and growing body of literature regarding gender differences in otolaryngology through examination of Canadian trends. Publication in peer-reviewed journals is the goal of scientific research and presentation at national conferences has important implications for clinicians’ careers. This research allows for identification of areas where gender disparities remain high to allow for solutions to address such disparities.

The number of women in otolaryngology in Canada has increased by 10% from 2000 to 2019 [3], but women are still compensated less [21], underrepresented in journal editorial boards [22], and have lower research productivity [23]. This study’s findings suggest that despite this gap, time to publication after presentation and the impact factor of journal of publication did not differ between the sexes. Women are publishing at similar rates compared to their male counterparts after podium and Poliquin presentations at CSOHNS meetings. The same is not true for female authors giving poster presentations as the publication rates were significantly lower for female first and senior authors. What accounts for the discrepancy between publication rates after poster, podium, and Poliquin presentation is unknown. Overall, male senior authors had higher rates of publication compared to female senior authors.

While it is encouraging that females are publishing at the same rate post-podium presentations, the similarity in the publication rate after Poliquin resident competition presentations may have a different interpretation. Given that the project must be at a stage of near completion, it is likely that submitted abstracts will be published, which could account for the similar publication rates of first male and female authors participating in this competition. Females represent 41.9% of OHNS trainees in Canada [3] but accounted for only 27% of Poliquin presentations, indicating that despite relatively similar publication rates, there is a disparity in female resident representation at national meetings.

While women comprised only 31.3% and 15.8% of first and senior authors in presentations, respectively, female representation within otolaryngology must be considered. In Canada in 2019, women accounted 38.9% of otolaryngologists in academic medicine, and 18.7% of academic faculty [3]. Chen et al.’s review of the gender landscape of otolaryngology showed a slow but consistent annual increase in the proportion of Canadian female otolaryngologists in all years studied [3]. The proportion of papers with female senior authors in this study, however, was lower than the current female representation within academia. These findings are supported by studies investigating h-indices in otolaryngology, a metric of scholarly productivity. Women have significantly lower h-indices, fewer publications, lower scholarly performance, and are less likely to be professors and hold senior academic roles [14, 24].

Given the increase in the number of women in otolaryngology and academic medicine, it is also expected that academic productivity after presentations at national meetings would increase. However, we found that the publication rate for females presenting at CSOHNS did not significantly change between 2009 and 2020. Many contributing institutional and cultural factors may account for this gender inequity and ongoing discrepancies in research productivity. At Canadian otolaryngology meetings, the main barrier to bringing manuscripts to publication was that the research was still in progress, which could indicate limited time for research [15]. Female senior authors were found to have significantly lower publication rates compared to men, which could be explained by the fact that women have less protected time to conduct research and receive less research funding [12, 25, 26]. Increased resources, such as more personnel to help coordinate projects and more time, may result in a greater rate of project completion [15].

Female first authors of otolaryngology papers are more likely to be non-physicians, such as junior trainees and medical students, and as such, they are also more likely to also be the presenter of posters at conferences [27]. Female medical students are also less likely to have a mentor compared to their male colleagues across varying levels of training [28]. Without adequate mentorship, completion of research may be difficult for females, possibly accounting for the gender differences seen in publication rates after poster presentation. Even after medical school, gender differences in mentorship persist in residents and staff physicians [28]. A lack of mentorship and women in leadership positions may limit visible role models, and act as a barrier to career development [29–32]. Mentorship is a critical aspect of academic medicine and increases research productivity, faculty retention, and improves well-being [33]. In addition, sociocultural barriers corresponding to family responsibilities, maternity leave, gender-based pay differentials, and work-place discrimination may also contribute to differences in research productivity, but reasons for these trends are currently unknown.

While systemic barriers limit women’s advancements within academic medicine, there have been improvements in the gender gap in otolaryngology. Between 2000 and 2015, female first authorship nearly doubled and female senior authorship marginally increased within otolaryngology literature [34]. While prior studies have shown that women lag behind men in the early career phase in terms of research productivity, recent literature shows that female otolaryngologists in certain subspecialities have similar publication productivity even in the early career time frame [14]. At a senior level, women are equal or exceed the research productivity of their male counterparts [23]. It has been hypothesized that this trend may be a result of evolving perspectives of gender roles and family units enabling equal sharing of childcare and domestic responsibilities which allow women to pursue full-time work [14]. Other possible factors that allow women to maintain their presence in academic medicine include women postponing child bearing to later years, shifts in parental attitudes towards utilization of daycare services, and the favoring a two-income households [35, 36].

Historically, pediatric otolaryngology has been the dominant subspeciality for female first author publication [37]. While females were more likely to publish in pediatric otolaryngology compared to men, head and neck was the dominant subspeciality of publication for both female first and senior authors. The difference in publication rate seen in this study is not due to subspecialty alone, however, as there was not a difference in likelihood of publication by each subspecialty without considering gender as a variable. Further supporting female advancement, a study compared female first author publications over time and found increases in first author publications throughout all otolaryngology subspecialties [27]. In more recent years, there is more balanced representation and contributions to literature from females in otolaryngology subspecialities [27].

This study has several limitations. Firstly, a binary definition of gender was utilized, while it is known that gender exists on a continuum. The assumption of the author’s gender was made based on their first name and images provided on faculty websites, and that may not have correctly identified an individual’s gender identity or personal pronouns. Secondly, conference abstracts were assessed until 2020, and it is possible that some abstracts are still undergoing publication. Studies have found that 90% or more of studies are often published within 4 years of a conference [38, 39], meaning that abstracts presented in the latter years of the analysis that are in the process of publication could have been missed, leading to an underreporting of publication rates. Thirdly, quality control by a fourth reviewer revealed that 2.7% of published articles were missed by reviewers. However, this is not a significant miss-rate to create a difference in the results.

## Conclusion

Gender differences exist in publication rates following presentation at Canadian otolaryngology conferences. The status of women in otolaryngology must continue to be monitored to ensure a diverse and equitable academic medicine workforce. The question remains what steps will be taken to address gender disparities that have limited opportunities for women in academic medicine and to support women’s leadership and academic research endeavors with otolaryngology. Improving female trainee mentorship and addressing institutional barriers may have a positive impact on research productivity and retaining women in academic medicine. The CSOHNS has taken a step in the right direction and has a branch dedicated to promoting women in otolaryngology and creating networking opportunities. Further emphasis should be placed on the advancement of women in fields such as research and grant funding.

## Data Availability

The datasets used and analysed during the current study are publicly available and can be found at https://www.entcanada.org/news-events/annual-meetings/.

## Abbreviations

OHNS: Otolaryngology–Head and Neck Surgery
AAO-HNS: American Academy of Otolaryngology–Head and Neck Surgery
CSO-HNS: Canadian Society of Otolaryngology–Head and Neck Surgery
